# Efficacy of topical clotrimazole vs. topical tolnaftate in the treatment of otomycosis. A randomized controlled clinical trial^☆^

**DOI:** 10.1016/j.bjorl.2018.12.007

**Published:** 2019-02-18

**Authors:** Lesly Jimenez-Garcia, Erika Celis-Aguilar, Gaudencio Díaz-Pavón, Victor Muñoz Estrada, Ángel Castro-Urquizo, Nemiliztli Hernández-Castillo, Ernesto Amaro-Flores

**Affiliations:** a Universidad Autónoma de Sinaloa, Centro de Investigación y Docencia en Ciencias de la Salud, Otolaryngology Department, Culiacán, Mexico; b Universidad Autónoma de Sinaloa, Centro de Investigación y Docencia en Ciencias de la Salud, Mycology Department, Culiacán, Mexico

**Keywords:** Otomycosis, Antifungals, Topical tolnaftate, Clotrimazole cream, Otomicoses, Antifúngicos, Tolnaftato tópico, Clotrimazole (creme)

## Abstract

**Introduction:**

Otomycosis, an infection of the ear canal by fungi, is prevalent in hot and humid weather. Nevertheless, there is not sufficient evidence for the effectiveness of different topical antifungal treatments. Tolnaftate, is a topical antifungal agent described to be effective in the treatment of otomycosis. Currently there are not sufficient studies that prove its efficacy.

**Objectives:**

To compare the efficacy of clotrimazole and tolnaftate administration in the treatment of otomycosis.

**Material and methods:**

A controlled, randomized and open clinical trial included patients diagnosed with fungal external otitis who were treated with topical antifungals, randomized into two treatment groups: (1) clotrimazole cream; (2) tolnaftate solution. They were microscopically evaluated at one and two weeks of treatment to determine resolution of disease. Recurrence and complications were recorded. Demographic and clinical variables were collected and analyzed. Follow-up and final outcomes (absence of infection) were compared between groups.

**Results:**

Forty eight patients were included, 28 in the clotrimazole group and 20 in the tolnaftate group. Spring was the weather most commonly associated with otomycosis, while otic manipulation was the risk factor more common in both groups. Predominant symptoms were itching and otic fullness. Aspergillus niger organism was isolated most frequently. Treatment with clotrimazole resulted in 75% resolution vs 45% resolution with treatment with tolnaftate at one week of treatment (*p* = 0.007). The Tolnaftate treatment group demonstrated higher recurrence rates and treatment failures, 20% and 15% respectively.

**Conclusions:**

Clotrimazole cream treatment is more effective than tolnaftate for uncomplicated otomycosis. More studies are needed to corroborate our results.

## Resumo

**Introdução:**

Otomicose, uma infecção fúngica do canal auditivo externo, é prevalente em climas quentes e úmidos. No entanto, a literatura não apresenta evidências suficientes sobre os diferentes tratamentos antifúngicos tópicos. O tolnaftato é um antifúngico tópico descrito como eficaz no tratamento da otomicose; entretanto, sua eficácia não está suficientemente comprovada.

**Objetivo:**

Comparar a eficácia do uso de clotrimazol e tolnaftato no tratamento da otomicose.

**Material e método:**

Ensaio clínico controlado e randomizado; incluiu pacientes diagnosticados com otite externa fúngica tratados com antifúngicos tópicos, randomizados em dois grupos de tratamento: 1) clotrimazole (creme); 2) solução de tolnaftato. Eles foram avaliados microscopicamente uma e duas semanas após o início do tratamento para avaliar a resolução da doença. Recorrência e intercorrências foram registradas; além disso, as variáveis demográficas e clínicas foram coletadas e analisadas. Os dados do acompanhamento e desfechos finais (ausência de infecção) foram comparados entre os grupos.

**Resultados:**

O estudo incluiu 48 pacientes, 28 dos quais foram alocados ao grupo clotrimazole e 20 ao grupo tolnaftato. A primavera foi a estação mais comum; a manipulação foi o fator de risco mais comum em ambos os grupos. Os sintomas mais comuns foram coceira e plenitude auricular. *Aspergillus niger* foi o micro-organismo mais comumente isolado. Após uma semana, o tratamento com clotrimazol apresentou uma taxa de resolução de 75% vs. 45% com o tratamento com tolnaftato (*p* = 0,007). O tratamento com tolnaftato apresentou maiores taxas de recidiva e falhas: 20% e 15%, respectivamente.

**Conclusões:**

Em casos de otomicose não complicada, o uso de clotrimazol (creme) é mais eficaz do que o de tolnaftato. Mais estudos são necessários para corroborar os presentes resultados.

## Introduction

Otomycosis is a term used to describe the epithelial infection of the External Auditory Canal (EAC) caused by yeast and filamentous fungi,^1^, ^2^ which accounts for 9% of external otitis diagnoses. Fungi are usually found in the outer ear as colonizers because this surface contains the necessary requirements for their growth: proteins, carbohydrates, humidity, temperature and adequate Ph.^3^ Predisposing factors include residing in tropical and humid climates, the use of long-term antibiotic or steroid therapy, a weakened immune system, lack of hygiene, a working environment with exposure to dust, foreign bodies in the EAC, cleaning of the EAC with swabs, genetic factors, seborrheic dermatitis and the presence of cerumen, all of which favor the germination of the spores and conidia of the prevalent fungi.^2^, ^4^, ^5^, ^6^, ^7^, ^8^ Species of *Aspergillus* (60–90%), usually *A. niger*, and *Candida* species (10–40%) are the most commonly cultivated pathogens.^2^, ^3^, ^4^, ^5^, ^7^, ^8^

Careful debridement of the EAC is crucial to facilitate the elimination of the infectious organism and to allow topical medications to reach the target tissue. Topical treatment cures most cases, although recurrence rates are high.^5^ Many agents with different antifungal properties have been used with varying success rates, so there is no consensus on the most effective agent. The objective of this study is to compare the efficacy of the administration of topical antifungal medications, Clotrimazole cream vs. Tolnaftate solution, in the treatment of fungal otitis externa.

## Methods

A controlled, randomized, open clinical trial was conducted in the otorhinolaryngology and head and neck surgery department of a second level hospital center from March 2016 to July 2017. Patients diagnosed with clinical otomycosis (visualization compatible with fungal debris was done through microscopic examination) were assigned to one of two treatment groups according to a computer-generated randomization table. Demographic and clinical data were collected, and two samples of affected EAC were taken from each patient with the help of an ear pick and/or suction cleaner and then placed in a sterile transport medium for direct microscopic examination and culturing in order to identify the pathogenic fungi involved. For the direct examination, the material was mixed with saline solution on a slide, covered with a coverslip and visualized under the optical microscope at 10× and 40× magnifications. For the culture, the sample was implanted in a Saboraud dextrose agar medium incubated at 27–30 °C for a minimum of 7 days. All patients underwent EAC cleaning and debridement. Clotrimazole cream was applied to the patients in Group 1 and left for 7 days; after that time, cream residue was removed from the EAC, and the otic conditions were re-evaluated. Patients in Group 2 were instructed to apply Tolnaftate solution, 2 drops every 12 h for 7 days, after which time the otic conditions were re-evaluated. The ideal clinical final outcome consisted of an asymptomatic patient with clean and dry external auditory canal confirmed through microscopic examination. In both groups, if the infection continued, the EAC was cleaned again and a second treatment with the same drug was administered. Patients who presented infection data after two courses of treatment were switched to the drug used by the other group. Dry ear care and avoidance of identified predisposing factors were advised. The degree of improvement was evaluated by comparing the symptoms and findings in the physical examination every week as well as one week after the resolution of the infectious disease to assess resolution or relapse. Since drugs in the imidazole group have been found in multiple studies to be effective in the treatment of otomycosis, clotrimazole was used as the standard of care.

The study was submitted for evaluation and approval by the Ethics Committee of our hospital with number 0149, and in all cases, an informed consent form was signed. This research adheres to the Declaration of Helsinki of the World Medical Association.

The data collected were entered into an SPSS database, demographic variables and baseline characteristics were analyzed, including measures of central tendency and data dispersion, analysis of categorical variables using Chi square and comparison of continuous variables with Student's *t*-test. A *p* < 0.05 was considered significant. Intention to treat analysis was performed; lost follow-up cases were considered failure cases, as long as one follow-up visit was undertaken.

A sample size of 25 patients was calculated in order to find equivalence between medications. Twenty five patients were determined in sample size calculation, with a power of 90%. Difference of effect between medications was estimated as −0.05 and 0.15, clotrimazol difference was assumed in 0.5, and calculations were made through a *Z* test and a significance of 0.05.

## Results

**Figure 1:**
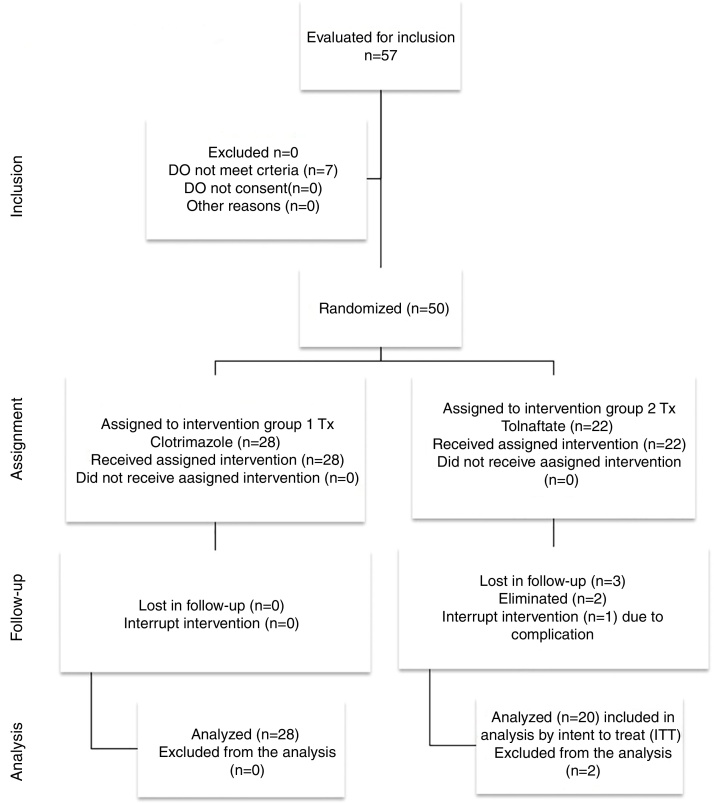
Flow chart of the study.

Figure 1 presents the flow diagram of the present study. Of the 48 patients studied, 28 patients were randomly selected to be included in Group 1 (Clotrimazole) and 20 in Group 2 (Tolnaftate) according to the computer randomization program. Table 1 shows the demographic characteristics and risk factors found in each group. Of the total 48 patients, 30 (62.5%) were male and 18 (37.5%) were female, with a male/female ratio of 1.6:1. Ages ranged from 12 to 77 years with an average of 41.70 ± 17.44 years. The most affected age group was 50–59, which represented 20.83% (*n* = 10) of all patients. The Clotrimazole group had more male patients than the Tolnaftate group (*p* = 0.034).

**Table 1:** Baseline demographic characteristics and risk factors in Clotrimazole and Tolnaftate groups.

Demographic characteristics	Group 1 Clotrimazole (*n* = 28)	Group 2 Tolnaftate (*n* = 20)	*p*-value
Gender (male)	21 (75%)	9 (45%)	0.034
Age in years (average)	42.75	40.25	0.665
Diabetic	2 (7.14%)	0	0.222
Use of earphones	7 (25%)	7 (35%)	0.452
Use of hearing aid	1 (3.5%)	1 (5%)	0.807
Ear manipulation with objects	23 (82.14%)	14 (70%)	0.324
Swimming	1 (3.5%)	0	0.393
Place of residence with unpaved streets	16 (57.14%)	14 (70%)	0.324

Two patients were eliminated. One patient was eliminated due to a complication at first week of treatment and evidence of a bacterial infection. The other patient did not complete the first follow-up visit.

The main occupations of the patients were housewives 18.75% (*n* = 9) and students 18.75% (*n* = 9), which was non-significant between the groups of treatment (*p* = 0.892). In both treatment groups, the majority of patients reported living in the city, representing 91.6% of the cases (*n* = 44). Overall, spring was the season with the highest infection rate, with 43.75% (*n* = 21) of the cases, followed by winter, with 27.08% (*n* = 13).

**Figure 2:**
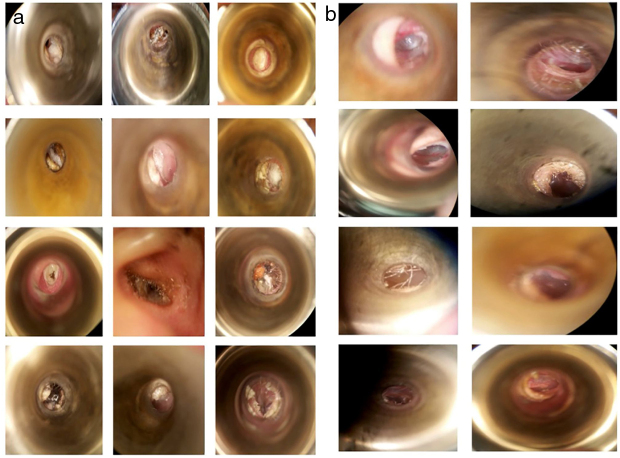
(a) Different types of hyphaes infecting the external auditory canal. Examples of microscopic view of patients in this study at time of inclusion. (b) Microscopic visualization after treatment. No hyphae are found or pathology in the external auditory canal.

The most frequent symptoms were pruritus 77.08% (*n* = 37), otic fullness 72.91% (*n* = 35) and hearing loss 62.5% (*n* = 30). There were no significant differences between the groups in the distribution of the initial symptoms (Table 2). The physical examination findings of the EAC (Fig. 2a) showed obstruction by ceruminous material, white 85.4% (*n* = 41), black 10% (*n* = 5) and yellow hyphae 2% (*n* = 1), and abundant desquamation 2% (*n* = 1).

**Table 2:** Clinical characteristics in Clotrimazole and Tolnaftate groups.

Clinical characteristics	Group 1 Clotrimazole (*n* = 28)	Group 2 Tolnaftate (*n* = 22)	*p*-value
Season when symptoms appeared (spring)	10 (35.7%)	11 (55%)	0.397
Average evolution time (days)	82.89	96.15	0.318
Affected side (right)	13 (46.4%)	10 (50%)	0.316
Pain	18 (64.2%)	11 (55%)	0.517
Hearing loss	18 (64.2%)	12 (60%)	0.762
Pruritus	19 (67.8%)	18 (90%)	0.072
Otorrhea	5 (17.8%)	3 (15%)	0.793
Tinnitus	10 (35.7%)	10 (50%)	0.322
Optical fullness	22 (78.5%)	13 (65%)	0.297
Vertigo	6 (21.4%)	7 (35%)	0.297
Other symptoms	2 (7.14%)	0	0.222

The direct exam mycological study of the samples was positive in 95.8% (*n* = 46) of the cases and showed that 45.8% (*n* = 22) of the patients presented mycelial fungal structures and 41.6% (*n* = 20) of the patients presented characteristics of *Aspergillus*. These data are summarized in Table 3. In the microscopic study of the cultures, the genus most frequently found were *Aspergillus*, 91.6% (*n* = 44) (Table 4).

**Table 3:** Sample findings from direct microscopic examination in Clotrimazol Group, Tolnaftate Group and total.

Direct exam microscopic findings	Total	Group 1 Clotrimazole	Group 2 Tolnaftate	*p*-value
*Aspergillus*	20 (41.6%)	15 (53.5%)	5 (25%)	
Mycelium	22 (45.8%)	11 (39.2%)	11 (55%)	
*Alternaria*	1 (2.08%)	1 (3.57%)	0	
Filaments	1 (2.08%)	1 (3.57%)	0	
Hyaline hypha spotted	1 (2.08%)	0	1 (5%)	
Microconidia	1 (2.08%)	0	1 (5%)	
Negative	2 (4.16%)	0	2 (10%)	
Total	48 (100%)	28 (100%)	22 (100%)	0.127

**Table 4:** Organisms isolated in cultures in Clotrimazole group, Tolnaftate group and total.

	Total	Group 1 Clotrimazole	Group 2 Tolnaftate	*p*-value
*Aspergillus*	44 (91.6%)	25 (89%)	19 (95%)	
*A. niger*	16	8 (28.5%)	8 (40%)	
*A. flavus*	9	5 (17.8%)	4 (20%)	
*A. terreous*	8	5 (17.8%)	3 (15%)	
*A. nidulans*	8	6 (21.4%)	2 (10%)	
*A. fumigatus*	3	1 (3.57%)	2 (10%)	
*Candida*	2 (4%)	2 (7.14%)	0	
No growth	2 (4%)	1 (3.57%)	1 (5%)	
Total	48 (100%)	28 (100%)	20 (100%)	0.715

Regarding the results of the treatment (Table 5), the infections of patients treated with clotrimazole cream completely resolved with one week of treatment in 75% of the cases (*n* = 21), while in the Tolnaftate group, resolution only occurred in 45% of the patients (*n* = 9). In both groups, the infection resolution after the first and second weeks of treatment showed a statistically significant difference, with *p*-values of 0.007 and 0.009, respectively.

**Table 5:** Comparison of treatment results in patients with Clotrimazol or Tolnaftate treatments.

	Group 1 Clotrimazole (*n* = 28)	Group 2 Tolnaftate (*n* = 20)	*p*-value
Resolution after 1st week of treatment	21 (75%)	9 (45%)	0.007
Additional patients with resolution after 2nd week of treatment	5 (17.8%)	7 (35%)	0.009
Recurrence	2 (7.14%)	4 (20%)	0.117
Number of recurrences	1 (2)^a^	1(3)^b^	0.220
		2 (1)^c^	
Change in treatment	0	2 (10%)	0.103
Complications	0	1 (5%)	0.254

a The two patients in the Clotrimazole Group who presented recurrence only experienced it one time each. Of the 4 patients in Group 2 who presented recurrence.

b 3 had recurrence on one occasion.

c 1 presented it on two occasions.

Group 2 showed a 15% (*n* = 3) treatment failure rate, and in 5% (*n* = 1) of patients, the infection was not resolved in the first week of treatment and presented complications (tympanic perforation and bacterial overgrowth). However, despite inadequate follow up (*n* = 3), twenty patients in Group 2 were included in the analysis under the principle of the intention to treat.

In patients who continued to present clinical manifestations after the first week of treatment, the most prevalent symptom was pruritus with 41.66% (*n* = 20) (Table 5). The resolution was evaluated according to the disappearance of symptoms and the presentation of an EAC free of hyphae or clinical signs of infection under the microscope (Fig. 2b).

## Discussion

The treatment options for otomycosis are multiple, and some of its treatments do not have clear scientific support yet, as in the case of Tolnaftate. On the other hand, azoles have been reported by some studies to be very effective in the treatment of otomycosis. In the present study, it was decided to compare Tolnaftate against Clotrimazole to determine the efficacy of both medications in a controlled clinical trial.

Regarding the epidemiology of this disease, the prevalence of otomycosis is closely related to the geographical area; our weather has a warm, sub-humid climate and presents optimal climatological conditions for the growth of pathogenic fungi. Most studies on the etiology of otomycosis have been performed in areas of high heat and humidity in addition to dust.^4^, ^9^, ^10^, ^11^, ^12^, ^13^, ^14^, ^15^, ^16^, ^17^ The prevalence by gender varies with respect to different studies; in our study, males were the most affected (62.5%), with a ratio similar to that reported by Viswanatha^18^; this difference was statistically significant (*p* = 0.034). With regard to age groups, patients in their 50s were more affected, which coincides with the results of Viswanatha.^18^

The high prevalence of otomycosis in the summer has been reported by several authors;^19^, ^20^, ^21^ however, in this study, the highest incidence occurred in the spring in both groups, with no statistically significance.

Otomycosis is mainly reported as unilateral in immunocompetent patients,^22^ however, Prasad^8^ mentioned that 5% of cases are bilateral, which is similar to our findings of 6%. The most frequent signs and symptoms reported in the literature are pruritus, otalgia, otorrhea, otic fullness, hearing loss and tinnitus,^2^, ^6^, ^8^, ^20^, ^23^, ^24^ all of which were present in our patients without statistically significant differences between groups.

Generally, otomycosis diagnosis is based on clinical findings, however, in this study it was also confirmed by mycological laboratory findings. In the direct examination, 100% of the samples from Group 1 and 90% of the samples from Group 2 demonstrated fungal structures. Culture findings vary widely. For example, Hueso-Gutiérrez^25^ reported only 22.6% positive cultures, while other studies have achieved yields close to 79%, and in this study the yield was high, confirming the diagnosis in 96% of cases. Several studies report that the most frequently isolated fungi (genus) are *Aspergillus* and *Candida*, the most common species being *A. niger* and *C. albicans*.^2^, ^8^, ^9^, ^14^, ^16^, ^22^, ^26^ Araiza^27^ reported *A. flavus* as the most common pathogen in Mexico City. In our study, the most frequent genus was *Aspergillus*, 89.2% in Group 1 and 95% in Group 2. In both groups, the most common species was *A. niger*, corresponding to that reported by other studies in hot and humid regions.

Treating otomycosis is difficult due to high recurrence rates.^6^ Failure to respond to the initial treatment has been reported up to 13% by Ho^2^ and recurrences vary from 5% to 15%.^28^, ^29^

The treatment recommendations are to control predisposing factors, local debridement and the use of antifungal agents, which was done with our patients from the first day of assessment. During the first week of treatment, subjective characteristics were evaluated, with pruritus being the most frequent symptom in both groups, similar to that reported in the literature.

Regarding antifungals, the imidazole group showed an 80% resolution rate in the initial application with scant probability of recurrence according to the Malik study.^12^ On the other hand, Jackman named Clotrimazole as the most popular and effective treatment,^30^ and others have reported effectiveness rates of 50–100%.^3^, ^14^, ^16^, ^29^ These findings coincide with the results of this study, where 82.14% of the cases treated with Clotrimazole completely resolved after one week of treatment. In our study, only 7.14% of Clotrimazole patients presented recurrence, and the infection was resolved with one more week of treatment without requiring treatment change. Furthermore, the application of Clotrimazole was easier and less expensive for the patient because it was carried out in the doctor's office and the patient had a check-up every week.

Regarding Tolnaftate, it has been recommended for refractory cases^14^; however, our results showed that only 45% of cases resolved after one week of treatment, 20% presented recurrence, 10% required a change in treatment to Clotrimazole, which resolved the infection, and 5% of the cases presented complications. However, recurrences, change in treatment or complications were not statistically significant, probably due to the small sample of this study.

Furthermore, there was a significant difference between the two treatment groups in the resolution of otomycosis after the first and second weeks of treatment. The Tolnaftate treatment also required greater patient adherence because it was applied at home every 12 h for 7 days. The cream is probably more efficacious than the drops because it covers all the extension of the EAC skin and stays in contact with this surface for a longer time.

Limitations of these studies mainly were the lack of treatment blinding, since both patients and doctors were aware which treatment was assigned. The difficulty in blinding resided on different treatment presentations, since in one group medication cream was applied in the doctor's office and the other topical drops at home. Furthermore, the latter treatment required more compliance from the patient, which was assessed in subsequent patient visits. Since our sample is small, more randomized controlled trials are needed to corroborate our results.

On the other hand, the highlights of this research include the comparison of two treatment options for otomycosis scarcely described in previous literature, as well as a careful design of a randomized controlled trial.

According to the results of this research, weekly application of Clotrimazole is recommended in patients with uncomplicated otomycosis.

## Conclusions

Treatment with Clotrimazole cream is more effective than treatment with Tolnaftate solution for uncomplicated otomycosis. More studies are needed to corroborate our results.

## Conflicts of interest

The authors declare no conflicts of interest.
